# Chronic posterior fracture-dislocation of the shoulder: case report and a literature review

**DOI:** 10.11604/pamj.2020.36.275.25046

**Published:** 2020-08-13

**Authors:** Ahmed Daoudi, Najib Abdeljaouad, Hicham Yacoubi

**Affiliations:** 1 Trauma-Orthopedic Service B, Mohammed VI University Hospital Center, Oujda, Morocco

**Keywords:** Posterior fracture-dislocation, shoulder, reverse Hill-Sachs lesion

## Abstract

Posterior shoulder fracture-dislocation is a rare injury accounting for approximately 0.9% of shoulder fracture-dislocations and often misdiagnosed during the initial presentation to a physician. Though the reverse Hill-Sachs lesion is a common injury associated with posterior shoulder dislocation, the associated scapula fracture represents only 6% of the lesions associated with a posterior dislocation of the shoulder. We report the case of a neglected posterior shoulder dislocation with a reverse Hill-Sachs lesion treated by filling with an autologous graft associated with an extra articular fracture of the scapula fixed by a plate and a posterior bone end-stop because of the posterior instability. After two years of follow-up, the patient has no episode of dislocation and is satisfied with the functional result with a constant score of 68/100 points.

## Introduction

Posterior dislocation of the shoulder is a rare traumatic injury representing only 2 to 5% of all dislocations of the shoulder [1]. Even less frequent, the posterior fracture - dislocation represents 0.9% of the 1,500 fractures - dislocation of the shoulder according to Neer and Foster, occurring annually in 0.6/100,000 people [2,3]. Posterior dislocation is missed in about 60% of the cases at the initial presentation to a physician [4]. Hill and McLaughlin [5] reported an interval of 8 months from trauma until the diagnosis was made. If the dislocation lasts longer than 6 weeks, then it is considered as chronic [6]. We report the case of a neglected posterior shoulder dislocation associated with a reverse Hill-Sachs lesion and a scapula fracture in a 57-year-old patient.

## Patient and observation

**Figure 1:**
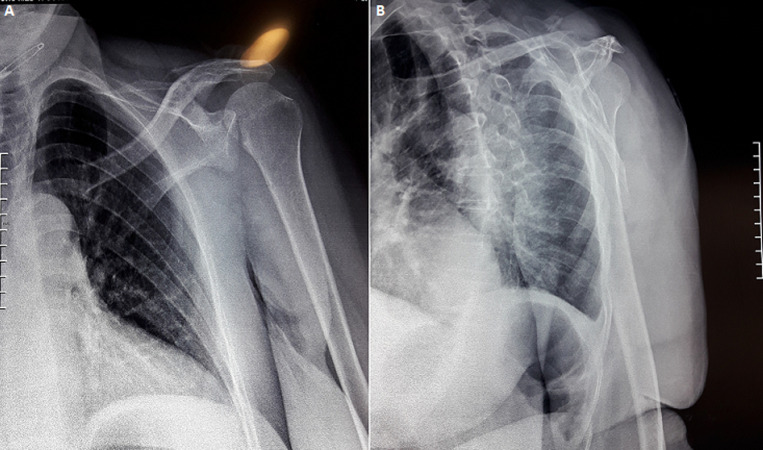
radiographs of the left shoulder: A) frontal radiograph of shoulder showing double contour of humeral head “trough sign” due to a reverse Hill-Sachs lesion; B) lateral view showing the humeral head projected behind the glenoid

**Figure 2:**
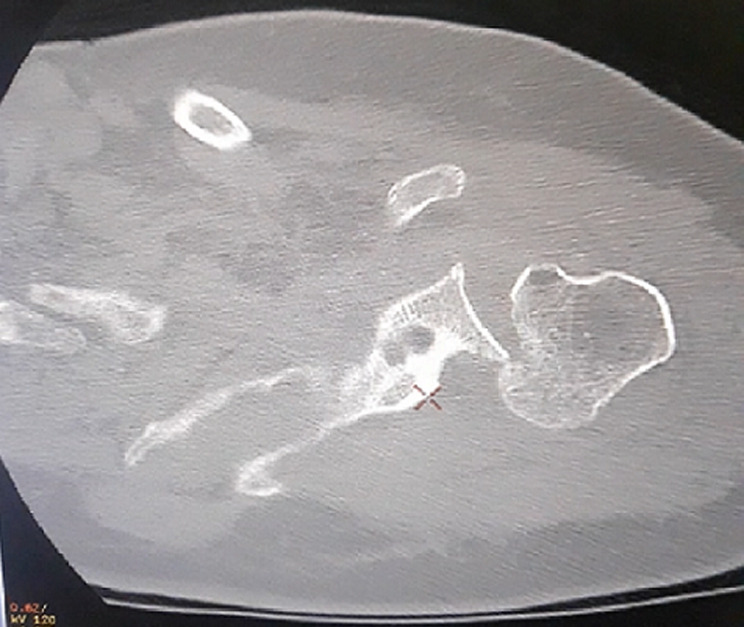
CT image showing a reverse Hill-Sachs lesion

**Figure 3:**
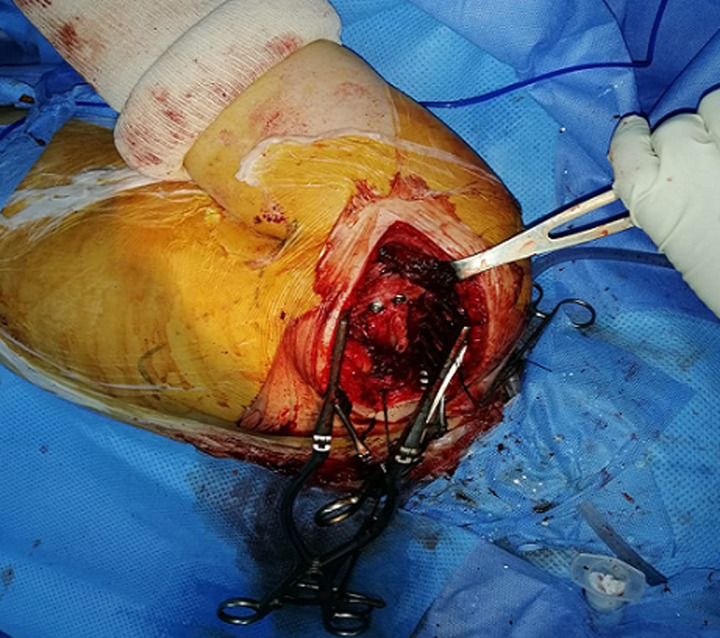
intraoperative image of posterior bone end-stop

**Figure 4:**
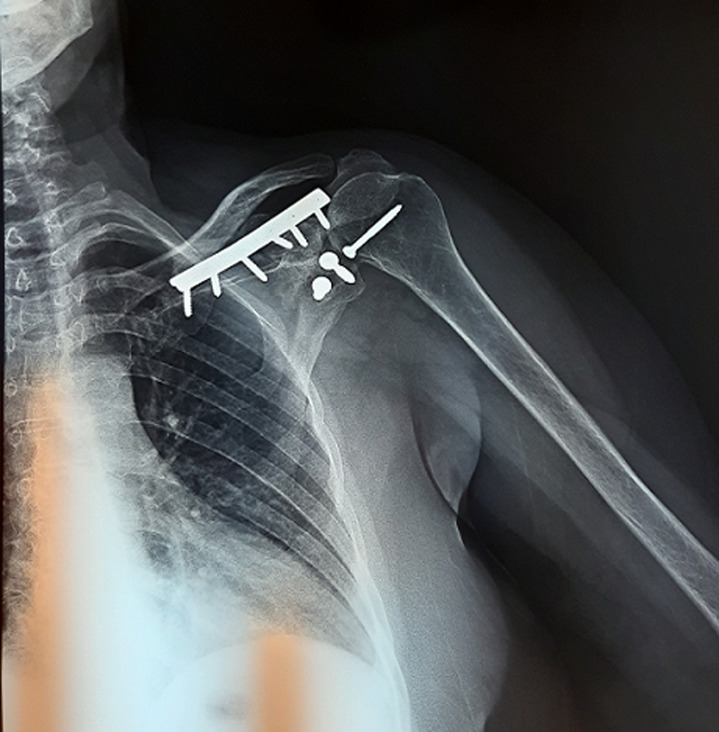
shoulder radiograph at 2 years showing no signs of osteoarthritis or osteonecrosis

This is a 57-year-old patient, without any particular pathological history and who was the victim of a fall from her height with a direct impact point on her left shoulder causing her major pain and functional impotence of her upper limb. Following a self-medication made of a simple scarf and medical treatment, the patient consults after 6 weeks of her trauma seen the non-improvement of symptoms. An initial clinical examination objectified a loss of the anatomical bony reliefs, a painful shoulder and blocked in attitude of adduction and internal rotation. Routine radiographs of the left shoulder (AP and lateral view) revealed no visible joint space. In the lateral view, the humeral head projected behind the glenoid as a sign of posterior shoulder dislocation (Figure 1). Closed reduction attempt under general anesthesia was performed without success. The computed tomography (Figure 2) allowed us to confirm the scapular fracture and the reverse Hill-Sachs. The size of this reverse Hill-Sachs defect was measured and expressed as a percentage of the projected total articular surface according to which it involved 30%. Under general anesthesia, the patient was installed in lateral decubitus with preparation of homolateral iliac crest. The approach was posterior subdeltoid, by an L-shaped incision along the spine of scapula. After an arthrotomy and joint approach, the filling of the reverse Hill-Sachs by an autologous graft fixed by a compression screw, the osteosynthesis of the scapula fracture by a plate and then a reduction of the glenoid joint. While passively moving the shoulder after an internal rotation, it came to redislocation of the humeral head in the posterior direction. We will proceed a third stabilization time by realizing a posterior bone end-stop by a tri-cortical graft which had been taken from the iliac crest fixed by two malleolar screws in compression (Figure 3). An additional immobilization in neutral rotation of the shoulder was recommended before starting the rehabilitation. At two years of follow-up (Figure 4), the patient is very satisfied with a constant score which has gone from 20/100 preoperatively to 68/100 currently.

## Discussion

**Figure 5:**
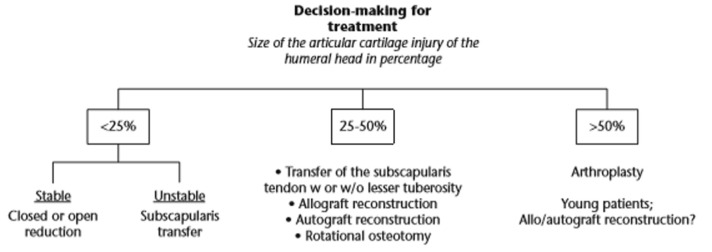
decision-making for the treatment of locked posterior shoulder dislocations

The posterior fracture - dislocation is a rare traumatic injury. Chronic posterior dislocations account for approximately 2% of all dislocations of the shoulder [7,8]. It is difficult to assess the prevalence accurately because more than two thirds of posterior dislocations are not recognized when they first present [8]. In a 2012 systematic review by Rouleau *et al*. impression fractures of the articular surface of the humeral head, the so-called reverse Hill-Sachs lesion, were the most commonly associated fracture type (29%) followed by humeral neck fractures (18.5%) and fractures of the lesser (14.3%) and grater (7.8%) tuberosity, while other fractures (humeral diaphysis, scapula, clavicle) were present in 6% of the cases [9]. The size of the reverse Hill-Sachs lesion is thought to be the most responsible factor for stability of the shoulder. They have been graded as small, when they affect up to 25% of the articular surface, medium, ranging between 25 and 50% and large when over 50% of the humeral head is impressed [10]. Instability of the shoulder thus determining the treatment plan, as defects over 25% demand operative intervention to restore stability [1,11,12]. Robinson *et al*. in their study of 26 patients with complex posterior fracture-dislocations treated operatively state that, when the head defect was restored intraoperative stability was achieved and addressing the posteroinferior capsulolabral avulsion was not required [3]. Surgical options are the elevation and supporting of the defect with cortico-cancellous bone chips, as performed in our case, the transfer of the lower tubercule (McLaughlin´s procedure) or the sub-scapularis tendon (Neer´s modified method) into the defect, sub capital rotational osteotomy (Weber´s procedure) of the proximal humeral head or arthroplasty [4]. Aydin N *et al*. [13] have proposed an algorithm for the management of the reverse Hill-Sachs lesion (Figure 5). Persistent instability after the restoration of the articular surface of the humeral head requires posterior stabilization. Posterior bone-block techniques were long, but necessary to restore stability and therefore a correct function [14]. At two years of follow-up, the patient does not show signs of osteoarthritis with a satisfactory functional result.

## Conclusion

The recognized options for the treatment of chronic posterior dislocation of the shoulder are dependent on the size of the anteromedial defect of the humeral head, the complexity of associated bones and ligaments damage, the degree of instability and the duration of dislocation.
